# Characteristics of Epstein-Barr virus-associated gastric cancer: A study of 235 cases at a comprehensive cancer center in U.S.A

**DOI:** 10.1186/1756-9966-28-14

**Published:** 2009-02-03

**Authors:** Camtu D Truong, Wei Feng, Wei Li, T Khoury, Q Li, S Alrawi, Yingyan Yu, Keping Xie, James Yao, Dongfeng Tan

**Affiliations:** 1 The University of Texas MD Anderson Cancer Center, Houston, TX, USA; 2 University of Texas Health Science Center, Houston, TX, USA; 3 Roswell Park Cancer Institute, Buffalo, NY, USA; 4 University of Florida at Jacksonville, Jacksonville, FL, USA; 5 Shanghai Tongji University School of Medicine, Shanghai, PR China

## Abstract

**Background:**

Epstein-Barr virus (EBV) has been shown to be associated with gastric cancer. However, inconsistent findings have been reported regarding the distribution of EBV infected cells (in normal gastric epithelium vs. intestinal metaplastic cells vs. in neoplastic cells) and the characteristics of EBV-associated gastric cancer. Lymph node positive EBV-associated gastric cancer has not been systematically studied. The aims of this study were to evaluate EBV-associated gastric cancer, to assess the distribution of EBV infected cells including all positive lymph nodes, and to define the characteristics of EBV-associated gastric cancer.

**Design:**

The study included primary gastric cancer patients who underwent surgical resection with no preoperative treatment at M.D. Anderson Cancer Center between 1987 and 2006. Formalin-fixed paraffin-embedded tissue from these resection specimens were assessed for EBV by in situ hybridization, the gold standard for EBV detection in tissue. EBV status was analyzed along with clinicopathologic parameters including age, gender, tumor type, lymph node status, and pathologic stage of the tumor.

**Results:**

Among 235 patients, 12 had intranuclear expression of EBV. EBV staining was seen only in tumor cells and no detectable EBV was observed in normal gastric mucosa, intestinal metaplasia or stromal cells. Eight of 12 patients with EBV-associated gastric cancer had regional lymph node metastasis. Of note, metastatic tumor cells in all of the involved lymph nodes of these 8 cases contained EBV. The epidemiologic data showed 11 of the 12 patients with EBV-associated gastric cancer were men, ranging in age from 54 to 78 years (mean age, 60 years; median age, 62.1 years). The age distribution for non-EBV associated gastric cancer patients ranged from 21 to 93 years (mean age, 67 years; median age, 66.4 years).

**Conclusion:**

Our study demonstrated that EBV is present exclusively in gastric cancer cells. The detection of EBV in tumor cells in all of the lymph nodes involved with metastatic gastric carcinoma suggests simultaneous replication of EBV and tumor cells. The predominantly male gender and relatively younger age observed for the EBV-infected gastric cancer cases suggest an association between this disease and other factors, such as life style.

## Background

In 1990, Burke et al. [1] used a polymerase chain reaction(PCR) method to detect Epstein-Barr virus (EBV) in a small group of gastric carcinoma cells that resembled cells of morphologically undifferentiated nasopharyngeal lymphoepithelioma. Subsequently, Shibata et al. [2], using in situ hybridization, demonstrated that EBV genomes were uniformly present in gastric carcinoma cells resembling lymphoepithelioma cells but were not present in reactive lymphoid infiltrate or normal mucosa. In addition, Shibata and Weiss [3] reported that EBV involvement was detected not only in lymphoepithelioma-like gastric carcinoma but also in a subset of ordinary gastric carcinomas.

During the past decade, the role of EBV in gastric carcinogenesis has been recognized as new evidences have continued to emerge [4-6]. EBV-associated gastric carcinoma (EBVaGC) harbors distinct chromosomal aberrations and is characterized by a unique transcription pattern that resembles but is not identical to that of nasopharyngeal carcinomas [7,8]. EBVaGC, compared with EBV-negative gastric carcinoma, shows distinct clinical features [9].

However, findings from studies in which various techniques were used to detect the presence of EBV in gastric cancer tissue have been highly controversial and conflicting. Some authors found EBV in only carcinoma cells [4,10-12], whereas others found EBV in both precursor lesions (e.g., intestinal metaplasia and dysplasia) and carcinoma cells [3,13-15]. Moreover, the status of EBV in the metastatic EBVaGC lymph nodes has not been investigated.

To further examine the role of EBV in gastric carcinogenesis, we systematically and retrospectively studied a large cohort of patients with gastric cancer in a single comprehensive cancer center using EBV-encoded RNA 1 (EBER1) in situ hybridization technique (the gold standard for identifying EBV, shown to be superior to EBV DNA in situ hybridization)[16]. We also utilized immunohistochemistry to detect EBV-specific proteins, which are known to be expressed in some EBV-associated malignancies [16].

## Materials and methods

### Patient population

For inclusion in this retrospective analysis, patients must have had a diagnosis of primary gastric carcinoma and undergone complete surgical resection of the tumor as initial treatment. The study criteria also included adequate archival tissue for analysis and the availability of complete clinicopathologic data. Patients who had received preoperative treatment (chemotherapy, radiotherapy, or chemo-radiotherapy) were excluded from the study. A total of 249 consecutive patients who had been treated at the University of Texas M. D. Anderson Cancer Center during the period of January 1, 1987 through December 31, 2006 met the study criteria.

The collected clinicopathologic data collected consisted of age, gender, date of initial diagnosis, tumor type, lymph node status, pathologic tumor stage, and date of death from gastric carcinoma or of last clinical follow-up. Histologic diagnosis and grade of differentiation were determined in accordance with the World Health Organization criteria for gastric tumors [17]. The M. D. Anderson Cancer Center institutional review board approval was granted to investigate molecular markers relevant to gastric cancer pathogenesis in this study.

### Histologic examination and tissue microarray construction

Hematoxylin and eosin-stained slides of gastric carcinoma tissue were reviewed to confirm the histopathologic diagnoses and to assess the adequacy of specimens before being selected for molecular analyses. We retrieved neutral buffered formalin-fixed (10% formalin in water, v/v; pH 7.4) and paraffin-embedded tissue blocks containing gastric carcinoma and nonneoplastic gastric tissue from the Department of Pathology at M. D. Anderson Cancer Center. One investigator (D.F.T.) identified and marked the areas containing viable tumor and normal tissue elements for the construction of tissue microarrays (TMAs). High-density TMAs were assembled using a tissue puncher-array system (Beecher Instruments, Silver Spring, MD), as we described previously [18]. Briefly, specimens retrieved from selected regions of archived donor tissue were precisely arrayed onto a new (recipient) paraffin block. Tissue cores were 1.0 mm in diameter and ranged in length from 1.0 to 3.0 mm, depending on the depth of tissue available in the donor block. For all cases, three tissue cores were acquired from each normal and tumor donor block. The three-core samples were subsequently inserted (spaced 0.8 mm apart) onto 45- × 20- × 12-mm recipient blocks. A total of four high-density TMAs were used in this study.

### In situ hybridization

To determine the localization of EBV in all specimens, we performed in situ hybridization using a digoxigenin-labeled 30 mer-oligonucleotide probe (EBER kit, Ventana Medical Systems, Tucson, AZ) (5' AGACACCGTCCTCACC ACCCGGGACTTGTA3') complementary to small nuclear EBER1, as described previously [19,20]. Briefly, 4-μm-thick sections were cut from paraffin-embedded tissues, mounted on slides coated with 3-(aminopropyl) triethoxysilane (Sigma Chemical Company, St. Louis, MO), baked at 60°C for 1 hour, and dewaxed. All sections were treated with 0.2 N HCl for 20 minutes, followed with 20 μg/ml proteinase K solution (Boehringer Mannheim, Mannheim, Germany). Next, the slides were dehydrated and prehybridized for 2 hours at 37°C with mixtures of 50% deionized formamide, 0.18 mol/l NaCl, 10 mmol/l NaH_2_PO_4_, 1 mmol/l ethylenediaminetetraacetic acid, 0.1% sodium dodecyl sulfate, 100 μg/ml of denatured salmon sperm DNA, 100 μg/ml of transfer RNA, and 10% dextran sulfate. The slides were then hybridized overnight at 37°C with 0.5 ng of digoxigenin-labeled probe. Follwed the first wash of all sections with 0.5 × saline sodium citrate, hybridization was detected by antidigoxigenin antibody-alkaline phosphatase conjugate. Next, all sections were subjected to a second wash follwed by a visualizing reaction performed with nitroblue tetrazolium salt and 5-bromo-4-chloro-3-indolyl phosphate solution in the dark for 6 to 12 hours. The slides were counterstained with methyl green and mounted with aqueous medium. Specimens from a patient with known EBV-positive gastric carcinoma were used as positive control, and a sense probe to EBER1 was used as negative control for each procedure.

### Immunohistochemical analysis

To detect EBV-specific proteins, which are known to be expressed in EBV-associated epithelial malignancies [16], we used monoclonal antibodies against latent membrane protein 1 (LMP-1). Serial 5-μm-thick tissue sections were cut from microarrays for immunohistochemical analysis. These sections were processed within 1 week of cutting to avoid oxidation of antigens. We stained the initial sections with hematoxylin and eosin to verify histologic type. We also used antigen retrieval and avidin-biotin staining and visualized the antibody with an avidin-biotin-horseradish peroxidase complex and diaminobenzidine-hydrogen peroxide staining method, as described previously by investigators from our laboratory [21,22]. Briefly, the sectioned array tissue was processed using steam-heat retrieval for 30 minutes. A mouse monoclonal antibody (CS1–4; Dako, Carpinteria, CA) against LMP-1 was reacted with the array sections for 25 minutes at room temperature in an automatic immunostainer (Dako). The array sections were then incubated in a detection kit in accordance with the manufacturer's instructions. Slides from the immunohistochemical analysis were independently reviewed by two investigators, who recorded the staining as negative or positive. All cells in all the cores were evaluated. Unequivocal nuclear staining in >5% of tumor cells was considered as positive response; nuclear staining in <5% of tumor cells was considered as negative response.

### Statistical analysis

The following variables were examined: age, gender, tumor type, lymph node status, pathologic stage, and EBV expression. For all statistical tests, two categories were analyzed in pairs as positive versus negative and present versus absent. We analyzed categorical variables using the Fisher's exact test, McNemar test and the Mann-Whitney rank-sum test. The follow-up time was calculated using the potential follow-up method. Overall patient survival was defined as the time between the date of surgical diagnosis to the date of last follow-up (censored) or the date of patient death (event). The end of follow-up date of this study was December 31, 2006. Censored cases included those cases (n = 6) in which the last follow-up date occurred before December 31, 2006. Patients who deceased of causes other than gastric cancer were not included in the study. We analyzed the differences in survival times between patient subgroups using the log-rank test. Survival probabilities were calculated (using the Kaplan-Meier method) and compared (using the log-rank test) [23].

We performed Cox proportional hazards regression analysis [24] using SAS software (SAS Institute, Cary, NC) to determine the association between the clinicopathologic variables and overall patient survival. First, we analyzed the association between possible prognostic factors (including age, gender, stage, and node classification) and death, considering one factor at a time. Second, multivariate Cox analysis was performed on backward (stepwise) procedures that always forced EBV into the model, along with all variables that satisfied an entry level of *P *< 0.05. As the model continued to add factors, independent factors did not exceed an exit level of *P *> 0.05.

## Results

### Clinicopathologic data

Clinicopathologic features of the study subjects are summarized in Table 1. Our study consisted of 88 female (37%), and 147(63%) male. One hundred eighteen (50%) patients were older than 65 years, while the other 117 (50%) were 65 years or younger. Eighty-three tumors (35%) were intestinal type, and 152 (65%) were diffuse type. One hundred thirty-one patients (56%) had stage I-II disease, and the remaining 104 patients (44%) had stage III or IV disease. Sixty patients (27%) had nodal involvement and 165 (73%) had no nodal metastases.

**Table 1:** Clinicopathologic features and EBV expression in gastric cancer

		**EBV Expression**			
		**Negative**	**Positive**	**Total**	**p**
**Gender**	Female	87 (37%)	1 (0%)	88 (37%)	0.03
	Male	136 (58%)	11 (5%)	147 (63%)	

**Age**	<65	111 (47%)	7 (3%)	118 (50%)	0.77
	> = 65	112 (48%)	5 (2%)	117 (50%)	

**Lymph node**	Negative	56 (25%)	4 (2%)	60 (27%)	0.74
	Positive	157 (70%)	8 (4%)	165 (73%)	

**Type**	Well/Moderately	79 (34%)	4 (2%)	83 (35%)	0.16
	Poorly	144 (62%)	8 (4%)	152 (65%)	

**Stage**	I or II	126 (54%)	5 (2%)	131 (56%)	0.38
	III or IV	97 (41%)	7 (3%)	104 (44%)	

**Total**		223 (95%)	12 (5%)	235 (100%)	

### EBV RNA expression in gastric tissue

We tested 249 gastric carcinoma tissues. Of the 249 tumor specimens, 235 were fully assessable. The yield after tissue processing was 94% (235 of 249). Among the 235 tumor cases, 72 also contained non-neoplastic gastric tissue (9 cases from EBV positive tumor cases and 63 from EBV negative cases). EBER1 was detected by in situ hybridization. Positive control samples revealed a distinctive diffuse nuclear stain. Sections incubated with preabsorbed or preimmune rabbit antisera showed no immunostaining.

**Figure 1:**
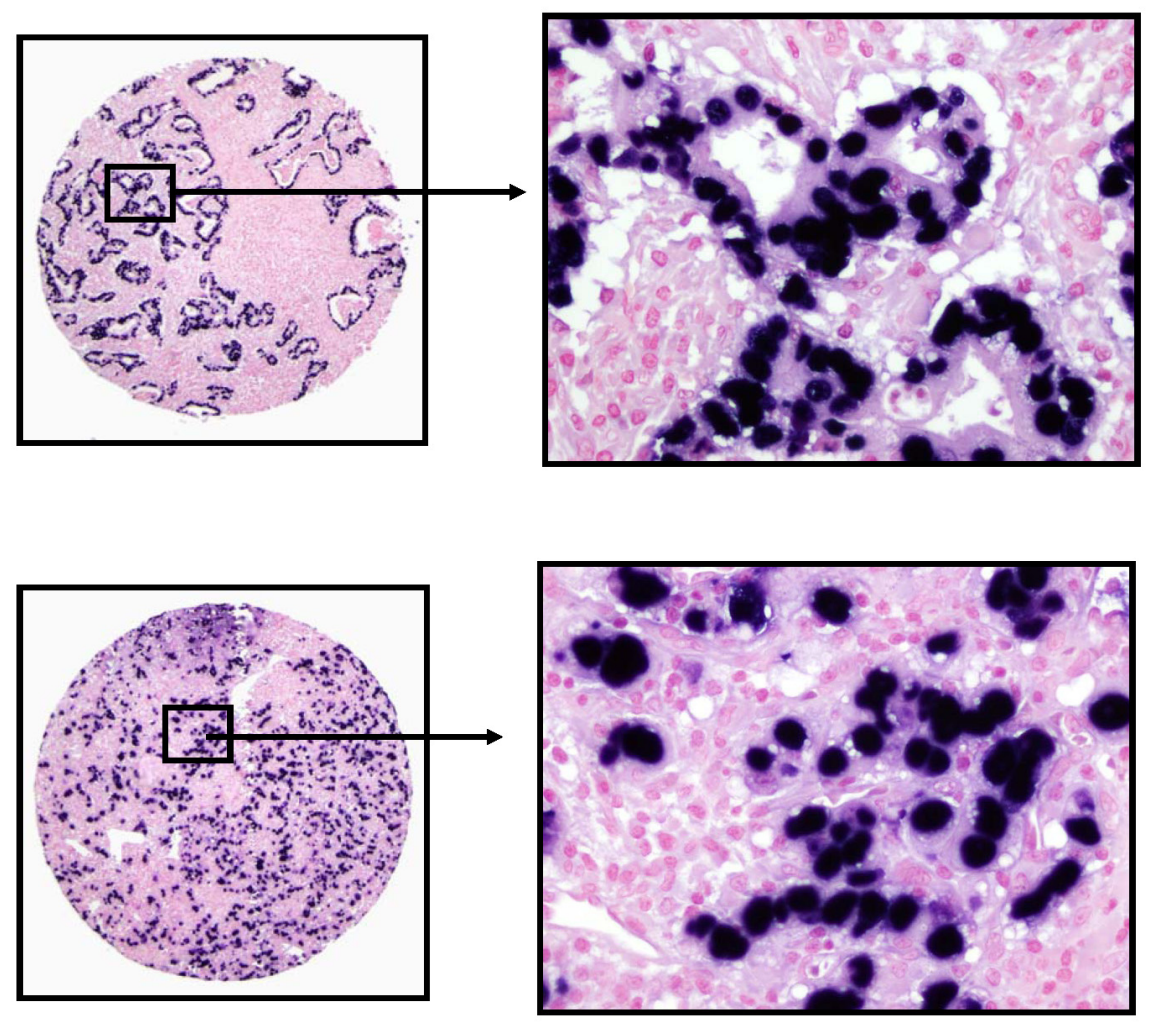
**Photomicrographs of Epstein-Barr virus (EBV) expression in gastric cancer**. Epstein-Barr virus (EBV)-encoded RNA 1 (EBER1) in situ hybridization in a gastric carcinoma reveals specific EBER1 transcripts (dark) in the nuclei of the tumor cells. 1A-B: intestinal type of gastric cancer with EBV nuclear expression. Note, all tumor glands were positive for EBV, while stromal cells between the tumor glands were negative. 1C-D: diffuse type of gastric cancer with EBV nuclear expression, while scattered lymphocytes were negative. (Original magnification × 10 in Fig. 1A&C, original magnification × 40 in Fig. 1 B&D)

**Figure 2:**
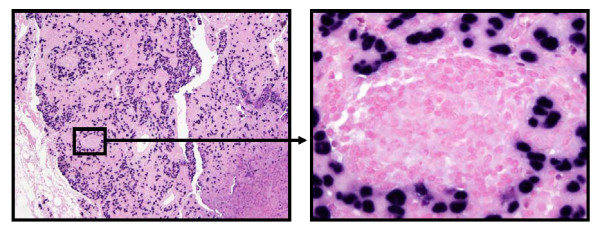
**A. Metastatic gastric adenocarcinoma involving lymph node (magnification × 10)**. 2B. Metastatic tumor cells are positive for EBV; germinal center is negative (magnification × 40).

Overall, 12 of the 235 tumors (5.1%) exhibited positive EBV expression (Figure 1). The intensity varied slightly from tumor to tumor but was consistent within the same tumor. No relationship was found between the intensity of EBER-1 expression and any clinicopathological features. EBV expression was noted in both diffuse (including lymphepithelial carcinoma) and intestinal type of GC (Table 1). Expression of EBV was not noted in nonneoplastic gastric mucosal, intestinal metaplastic, or stromal cells (endothelial cells and fibroblasts), or infiltrating inflammatory cells within the tumor sections. Twelve of 235 gastric tumor cases exhibited EBV expression, while none of the 72 samples containing non-neoplastic gastric epithelium displayed EBV expression. The difference between EBV positivity in carcinoma tissues and corresponding non-neoplastic gastric tissues was statistically significant (χ^2 ^= 9.0407; *P *= 0.0028). In addition, one representative positive lymph node from each metastatic case was examined. We observed that a fairly uniform expression of EBER1 in metastatic tumor cells. Among the 12 EBVaGC cases, eight patients displayed lymph node metastasis. Tumor cells in all eight positive lymph nodes revealed EBV expression (Figure 2). Ten additional metastatic cases were randomly chosen and lymph nodes with tumor cells were examined for EBER1. No tumor cells in the lymph nodes of the 10 additional cases displayed EBER1 expression.

### LMP-1 protein expression in gastric tissue

Positive control, using known LMP-1-positive lymphoid tissue, revealed a distinctive membranous stain. Negative control sections were immunostained under the same conditions, with preabosorbed antisera substituted for the primary antibody, displaying no immunoreactivity. Among all 249 tested, 231 were assessable. No expression of LMP-1 was identified in any gastric cancer or in non-neoplastic gastric tissue.

To verify the foregoing TMA results, we examined a subset of 40 whole tissue sections (from 12 patients with EBVaGC and 28 without EBV) for the expression of EBV and LMP. The findings were consistent with those from the TMA cores. EBV was detected only in the EBVaGC sections; no EBV was observed in nonneoplastic gastric tissue or in intestinal metaplasia.

### Association of EBV expression with clinicopathologic parameters

**Figure 3:**
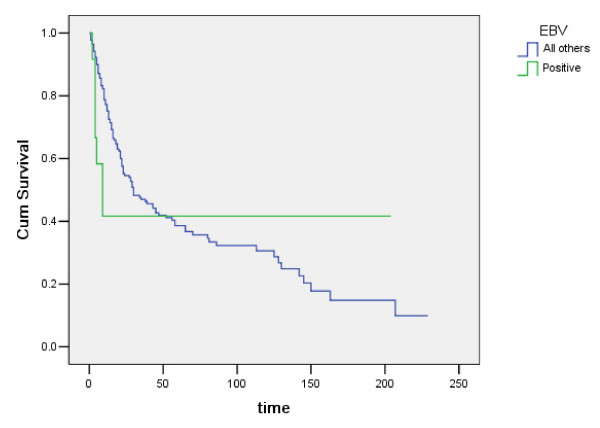
**Survival graph of EBV associated gastric cancer and non-EBV associated gastric cancer**.

Age, gender, tumor type, nodal status, and pathologic tumor stage were the clinicopathologic parameters analyzed in our study. After examining the associations between EBV expression and clinicopathologic variables (Table 2), we found a statistically significant association between EBV expression and gender. Eleven of the 12 patients with EBVaGC were male. The difference in EBV positivity in carcinoma tissues between male and female patients was significant (*P *< 0.05). Patients with EBVaGC were 54–78 years old (mean age, 60 years; median age, 62.1 years), whereas patients with gastric cancer not associated with EBV were 21–93 years old (mean age, 67 years; median age, 66.4 years). Subsequently, we analyzed the differences in survival times between patient subgroups using the log-rank test. Survival probabilities were calculated (using the Kaplan-Meier method) and compared (using the log-rank test). Compared to those without EBV expression, patients with EBVaGC displayed a favorable clinical outcome (Figure 3). However, by multivariate Cox analysis, only lymph node status and tumor stage were significantly associated with ultimate patient prognosis (Table 3).

**Table 2:** Association of EBV expression and clinicopathologic variables

**Univariate analysis**		**RR**	**95% C.I**.		
			**Lower**	**Upper**	**p**
**EBV**	Negative	1.00			
	Positive	1.52	0.71	3.27	0.28

**Gender**	Female	1.00			
	Male	0.96	0.68	1.36	0.83

**Age**	<65	1.00			
	> = 65	0.86	0.61	1.22	0.40

**Lymph node**	Negative	1.00			
	Positive	2.97	1.87	4.72	0.00

**Type**	Well/Moderately	1.00			
	Poorly	1.50	1.18	2.39	0.05

**Stage**	I or II	1.00			
	III or IV	2.14	1.51	3.03	0.00

**Table 3:** Multivariate analysis: Association of EBV, lymph node status and tumor stage of gastric cancer with patient's survival

**Multivariate analysis**		**RR**	**95% C.I**.		
			**Lower**	**Upper**	**p**
**EBV**	Negative	1.00			
	Positive	1.56	0.72	3.37	0.26

**Lymph node**	Negative	1.00			
	Positive	2.47	1.48	4.11	0.01

**Stage**	I or II	1.00			
	III or IV	1.49	1.01	2.20	0.04

## Discussion

Gastric carcinoma is one of the most common cancers worldwide and the second most common cause of cancer-related death, with 876,000 new cases diagnosed annually [17]. In addition, EBV-positive gastric cancer cases make up the largest group of EBV-associated malignancies. Thus, defining the role of EBV in the carcinogenesis of this widespread malignancy is essential.

Using in situ hybridization technique, we examined 235 cases of primary gastric cancers, which to our knowledge was the largest study group of this type in the United States. Specific nuclear EBER1 transcripts were found only in gastric carcinoma cells. In contrast, EBV was detected in none of the normal or dysplastic epithelia in the EBVaGC or EBV-negative cases. Specifically, in 10 of the 12 cases of EBVaGCs, EBER1 was expressed in almost all carcinoma cells, suggesting that EBV infection occurs early in oncogenesis with a subsequent clonal expansion of EBV-containing tumor cells, significant findings which have also been reported by investigators using molecular genetic techniques [13,25]. In two cases of EBVaGC, EBER1 was expressed in a small number of gastric carcinoma cells, visualized with focal EBER1 staining, indicating that EBV infection occurs after neoplastic transformation has taken place.

The EBV nuclear expression was restricted to gastric carcinoma cells. No expression was found in the presumed precursor lesions of gastric carcinoma. Our results agree with those of other studies in which EBER transcripts were not detected in adjacent precursor lesions, such as intestinal metaplasia [4,26-28]. However, some studies have described the presence of EBV in dysplasia [3,13], and others have detected the presence of EBV in intestinal metaplasia [14,15]. There are several reasons for these discrepancies. First, dysplasia adjacent to carcinomas is difficult to distinguish from local carcinoma spread [17]. Secondly, variation in the techniques used and methods of interpretation can lead to inconsistent results. For example, one study that used both polymerase chain reaction and in situ hybridization indicated that the EBV genome was detected by polymerase chain reaction in one case of normal gastric mucosa, but not by in situ hybridization [19].

Recently, one study examining EBV in gastric carcinomas and gastric stump carcinomas and found that EBER1/2 transcripts were restricted to the carcinoma cells in both types of cases [12,29]. The absence of EBER1 transcripts in preneoplastic gastric lesions (intestinal metaplasia and dysplasia) but their presence in two distinct types of gastric carcinoma further supports the theory that EBV can infect only neoplastic gastric cells.

Our study showed that LMP-1 expression was not found in EBV-positive carcinomas or their precursor lesions, which is in line with previous observations [28,30-32]. The absence of LMP-1 expression in EBVaGCs suggests that LMP-1 may not be necessary for such tumors, at least not for sustaining their already established malignant state. Rather, LMP-1 may participate in the earlier stage of tumor development and may be down-regulated thereafter. Alternatively, the lack of LMP-1 may reflect the result of clonal selection of LMP-1-negative tumor cells by immunologic pressure because EBV-specific cytotoxic T cells are potentially directed against the viral LMPs rather than against EBV nuclear antigen 1. Yanai et al. [15] reported that EBV-LMP-1 was observed in cases of atrophic gastric mucosa. However, this finding is not likely to be confirmed due to the inconsistent results from in situ hybridization and due to the fact that the researchers used a biotin method. It has been demonstrated that cross-reactivity can occur and that the interpretation of positive immunohistochemical results should always be done in the context of transcript analysis by reverse transcription polymerase chain reaction [7,28] and EBER1 in situ hybridization [4].

In this population, a 5.1% prevalence of EBV in gastric cancer was observed, comparable with the prevalence of EBV detected in gastric adenocarcinomas worldwide [4,25,33] and indicating that the overall prevalence of EBV in gastric carcinomas is independent of geographic regions [11,29]. Our observations of male predominance and younger patient age are in agreement with those of several previous studies [3,33,34]. However, ours was the first large study of this type conducted in the United States. Our male-to-female ratio of 9.2 was among the highest described so far. A male:female ratio of 9.8 was reported in one large cohort Dutch study [4].

In short, this study, evaluating the distribution of EBV infected cells in a large cohort of patients at a single comprehensive cancer center in U.S.A, confirms that EBV is restrictly expressed in tumor cells and predominately in younger male patients. Furthermore, positive EBV-infected tumor cells were observed in all lymph nodes with metastasis. The detection of EBV in metastatic tumor cells in all of the lymph nodes involved with gastric carcinoma suggests simultaneous replication of EBV and tumor cells. The predominantly male gender and relatively younger age observed in our study suggest an association between EBV-infected gastric cancer and other factors, such as life style.

## Competing interests

The authors declare that they have no competing interests.

## Authors' contributions

CDT and WF carried out the pathology review and data collection, data review, participated in study design and coordination. WL, TK, and SA participated in study design and drafting the manuscript. OL carried analyzing data. YY, KX, and JY participated in study design, data collection and coordination. DT was the principle investigation of the study and participated in all aspects of this work. All authors read and approved the final manuscript.
